# Examining the role of pre‐visit anxiety on patient uncertainty and breast cancer patient–provider communication

**DOI:** 10.1002/cam4.70003

**Published:** 2024-07-19

**Authors:** Elizabeth A. Broadbridge, Maria K. Venetis

**Affiliations:** ^1^ Department of Communication Rutgers University New Brunswick New Jersey USA

**Keywords:** anxiety, breast cancer, breast neoplasms/psychology, communication, communication barriers, oncology, patient participation, physician–patient relations, surveys and questionnaires, uncertainty

## Abstract

**Objective:**

Effective communication between cancer patients and providers is critical for addressing psychological distress, reducing uncertainty, and promoting patient well‐being. This is particularly relevant during medical appointments that may elicit uncertainty, such as surgical consultations for newly diagnosed women with breast cancer. This study aimed to evaluate how pre‐appointment anxiety and illness uncertainty affect patient–provider communication in breast cancer surgical consultations and subsequent post‐appointment well‐being. Breast cancer patient anxiety has been studied as an outcome of provider communication, though less is known about the extent to which preexisting anxiety or uncertainty act as antecedents to effective patient–provider communication.

**Methods:**

This study analyzed videorecorded breast cancer surgical consultations (*N* = 51) and corresponding patient surveys to understand how pre‐appointment anxiety influences pre‐appointment patient uncertainty, patient–provider communication during the appointment, and subsequent post‐appointment uncertainty.

**Results:**

The proposed model achieved good fit to the data such that more pre‐appointment anxiety was associated with more pre‐appointment uncertainty, more pre‐appointment anxiety was associated with more empathic opportunities per minute, and more empathic opportunities were associated with less post‐appointment uncertainty.

**Conclusions:**

Results indicate breast cancer patients with anxiety pre‐appointment are at‐risk for more illness uncertainty and are more likely to explicitly provide empathic opportunities. This supports the need for added attention to empathic opportunities to not only address patients emotionally but to also assess whether a patient may be at higher risk of having preexisting anxiety.

## Graphical abstract

This study analyzed videorecorded breast cancer surgical consultations (*N* = 51) and corresponding patient surveys to understand how pre‐appointment anxiety influences pre‐appointment patient uncertainty, patient–provider communication during the appointment, and subsequent post‐appointment uncertainty. We found that more pre‐appointment anxiety was associated with more pre‐appointment uncertainty, more pre‐appointment anxiety was associated with more empathic opportunities per minute, and more empathic opportunities were associated with less post‐appointment uncertainty.

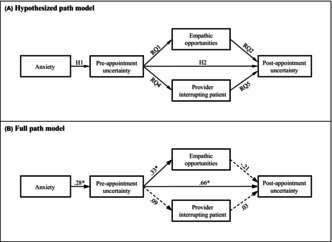

## 1 INTRODUCTION

Breast cancer accounts for more than one third of all cancer in women in the United States^1^ with an estimated 310,000 newly diagnosed women in 2024 alone.^2^ Anxiety is highly prevalent among breast cancer patients, and more than one third of patients in the United States experience a degree of anxiety throughout their diagnosis and treatment.^3^, ^4^ Importantly, breast cancer patients who have symptoms of anxiety have a higher risk for both morbidity and all‐cause mortality.^5^, ^6^ It is critical that cancer care providers have the tools to understand and address the high prevalence of anxiety in breast cancer patients to meet the needs of this population. Current research favors anxiety and psychological distress as outcomes of medical interactions.^7^, ^8^ However, given the high prevalence of anxiety in this patient population, it is important to investigate the ways in which preexisting levels of anxiety affect additional aspects of breast cancer patients' processing and coping with their diagnosis. One potential consequence of anxiety ahead of a medical appointment is patients' illness uncertainty. Using Uncertainty in Illness Theory (UIT)^9^ as the framework, this study explores how breast cancer patient anxiety and uncertainty pre‐appointment influence patient–provider communication and subsequent post‐appointment uncertainty.

### 1.1 Breast cancer and uncertainty

Illness uncertainty is a cognitive and emotional state in which patients dealing with novel, complex, and/or ambiguous health events are unable to make meaning of these events.^9^ Breast cancer patients experience this throughout the illness trajectory, from diagnosis to treatment and beyond.^10^, ^11^, ^12^ Uncertainty about the diagnosis, cancer‐related symptoms, illness prognosis, and treatment decisions all contribute to a worse quality of life for breast cancer patients.^13^, ^14^, ^15^ Moreover, illness uncertainty is positively correlated with anxiety during diagnosis^16^ and is associated with worse psychological well‐being during both the treatment and post‐treatment stages.^17^

**FIGURE 1:**
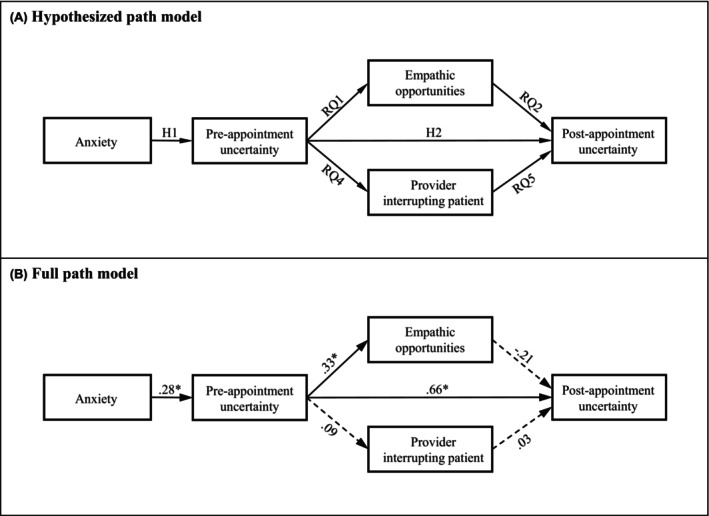
Hypothesized model of post‐appointment uncertainty (A) and results of the full path model for post‐appointment uncertainty (B). Parameter estimates are standardized. Solid lines in Figure 1A, B represent significant paths at *p* < 0.01 (designated with *). Dashed lines represent paths with significance at *p* > 0.05. (B) Model fit indices were *χ* ^2^ (4) = 4.29, *p* = 0.37; RMSEA = 0.04; CFI = 0.99; SRMR = 0.07.

UIT posits that illness uncertainty is influenced by patients' perceptions of illness‐related events (*stimuli frame*), and appraisal of the stimuli frame is influenced by patients' *cognitive capacity*.^9^, ^18^ UIT further suggests that appraisals of illness uncertainty subsequently influence coping with illness. Most investigations of UIT that measure psychological features have considered it in relation to coping and as a final outcome of the model,^17^, ^18^, ^19^, ^20^ but have not explored how cognitive capacity is posited to affect patient outcomes. This study seeks to further understand the cognitive capacity construct, investigating how anxiety acts as an independent variable in the model. Considering anxiety as the *cognitive capacity* construct, according to UIT patient anxiety would predict the level of uncertainty. Although anxiety and uncertainty in this context potentially have overlapping consequences for cognition,^16^ this study tests the relationship as a sequenced relationship outlined by UIT. We propose the following hypotheses (see Figure 1A for the hypothesized model):

Hypothesis 1 Anxiety will be positively associated with breast cancer patients' pre‐appointment illness uncertainty.

Hypothesis 2 Breast cancer patients' pre‐appointment illness uncertainty will be positively associated with post‐appointment illness uncertainty.

### 1.2 Anxiety, uncertainty, and patient communication

Goals of patient participation in medical encounters include patient information seeking and sharing. One form of patient information sharing includes *empathic opportunities* in which patients offer statements that express an emotion such as fear, worry, or relief and explicitly give medical providers opportunities to respond empathically and with support.^21^ Thus, this form of communication allows patients to share information and can elicit provider responses, contributing to the overall patient–provider relationship.

Patients with better psychological adjustment are often more participative than those experiencing maladaptive coping styles^22^; for example, patients with increased anxiety are more likely to be indirect when expressing their concerns than patients with less anxiety.^23^ Similarly, increased uncertainty is associated with cognitive states that bias information processing and subsequent communication.^24^ It is possible that patient anxiety and associated uncertainty may similarly bias information processing and, subsequently, influence patient communication. However, the connection between illness uncertainty and communication consequences in oncology appointments has not been investigated, and we ask:

How is pre‐appointment illness uncertainty associated with differing levels of breast cancer patient empathic opportunities?

#### 1.2.1 Provider communication: Interruptions and responses to empathic opportunities

Goals of high‐quality provider communication include facilitating patient participation within medical interactions^25^ and considers patients' psychological well‐being. Positive provider responses to patient empathic opportunities may help achieve this goal. For example, providers may affirm patient concerns or could alternatively dismiss the information shared.^26^ How providers respond to patient communication is consequential to patient well‐being, with expressions such as hopefulness associated with more satisfaction with care.^27^ Cancer care providers often miss these opportunities,^28^, ^29^ which may hinder patient comfort and participation.

Are empathic opportunities from breast cancer patients associated with differing levels of post‐appointment illness uncertainty?

Does the type of provider response to empathic opportunities predict levels of post‐appointment illness uncertainty?

Similarly, another provider communication pattern that could influence patient wellbeing is through provider conversational dominance, specifically, provider interruptions. When providers interrupt or talk over patients, patients may not have the opportunity to fully express their thoughts, feelings, and concerns, which can be in direct opposition to facilitating patient participation. Interruptions may constrain patients from receiving desired information, influencing their uncertainty. Thus, we anticipate that increased interruptions will negatively influence patient well‐being and will be associated with increased patient uncertainty. We also question if patient state, such as increased anxiety or uncertainty, impacts provider interruption behavior. To investigate the relationships between patient illness uncertainty, oncologist responses to empathic openings, and oncologist interruptions, the following research questions are proposed:

How is patient pre‐appointment illness uncertainty associated with differing levels of interruptions by the oncologist?

How are oncologist interruptions associated with differing levels of post‐appointment illness uncertainty?

## 2 METHODS

### 2.1 Sampling and procedures

This study involved secondary analysis of videorecorded breast cancer surgical consultations and patient surveys.^30^ Primary data were collected between April and December 2009 at a National Cancer Institute‐designated comprehensive cancer center in the northeastern United States. Eligible participants were English‐speaking women newly diagnosed with breast cancer attending a surgical consultation. Consultations included a physical examination and a discussion of surgical options. Following the examination, patients dressed and were then moved to a nearby room. A researcher approached patients and their companions for participation after they arrived in the consultation room and before the surgeon returned. All newly diagnosed women with breast cancer who had been informed of their diagnosis were approached for recruitment, and 74/88 (84%) consented to participate. A single breast surgeon conducted all consultations. Of the 74 patients who consented to be part of the study, 51 completed both pre‐ and post‐appointment surveys. Video recordings of each consultation were coded by the first author and a subset (10%) of the recordings were coded by the second author to establish inter‐rater reliability using Cohen's Kappa (*κ* = 0.78; *p* < 0.01). Participants were compensated $20. The original study was approved by the Rutgers University Institutional Review Board.

### 2.2 Measures

#### 2.2.1 Anxiety

Anxiety (H1) was measured using the 7‐item anxiety scale from the Hospital Anxiety and Depression Scale (HADS).^31^ The HADS asked participants to rate how often they experienced symptoms of anxiety on 4‐point scales specific to each item. An example item is, “I feel tense or ‘wound up’” (*not at all* to *most of the time*). The scale achieved good reliability (*α* = 0.89). Items were totaled for a score that could range from 0 to 21. Scores from 0 to 7 are considered no anxiety, 8–10 are borderline anxiety, and scores ≥11 indicate symptoms of anxiety.^31^ Scores were dichotomized as anxiety (HADS ≥11, *n* = 12) and low anxiety (HADS <11, *n* = 35) for group comparisons across demographic variables and left as a continuous ordinal score for modeling.

#### 2.2.2 Pre‐ and post‐appointment uncertainty

Illness uncertainty (H1 and H2, [Link], [Link], [Link], [Link], [Link]) was assessed using an adapted 7‐item version of the Uncertainty in Illness Scales.^32^ Participants responded to the scale pre‐ and post‐appointment. Items were adapted for this population and treated based on factor analyses described previously.^33^ Participants rated the degree to which they agreed with statements about their uncertainty on a 5‐point scale (*strongly disagree* to *strongly agree*). An example item is, “Because of the unpredictability of my illness, I cannot plan for the future.” Pre‐ and post‐appointment uncertainty achieved good reliability (*α* = 0.86 and 0.80, respectively). Items were summed for a final composite score that could range from 7 to 35. Higher scores indicate more illness uncertainty.

#### 2.2.3 Empathic opportunities

Empathic opportunities (RQ1 and RQ2) were assessed using an adapted form of an established empathic communication coding framework.^21^ Empathic opportunities were defined as statements that directly expressed an emotion such as fear, worry, or relief. In a limited number of cases (*n* = 3) the provider initiated an opening by inviting the patient to share their feelings without verbal prompting by the patient. Most consultations (68.63%) had between one and three empathic opportunities. Two appointments had no empathic opportunities and one appointment had nine opportunities. To account for varying lengths of appointments, number of empathic opportunities was divided by length of the consultations for a rate of empathic opportunities.

To address RQ3, provider responses to each empathic opening were also coded in alignment with the above framework.^21^ Responses were coded as missed, positive, or negative (see Table 1). Missed opportunities were those where the provider did not explicitly acknowledge the emotion behind the statement and continued with the discussion. Responses that acknowledged the empathic opening were further categorized as either positive or negative. Provider‐initiated empathic openings were coded as positive empathic openings. To account for varying lengths of appointments, the total number of each response type was divided by consultation length for response type rates.

**TABLE 1:** Example empathic opportunities and responses.

	Example
Patient‐initiated opening *n* = 147	P: So that is going to be a separate issue altogether? O: Yes. I doubt that it is going to be an issue for you, and I am just basing that on experience. P: I'm just trying to prepare myself, you know, asking the worst questions so this way I am not worrying with it.

Possible responses	Example
Missed opportunity *n* = 96 (64%)	O: So let's assume that it is okay and so we don't really have to do anything over there, just deal with the left side. P: We can be hopeful. I could use something good. O: So at that point we've done the mastectomy and we've done the reconstruction so the treatment of the breast is done. Now, we're going to totally switch gears now.
Positive response *n* = 25 (17%)	P: …I think the thing that stresses me and gets me worried and panicked is surgery. I never had surgery. I had my wisdom teeth removed, you know, but that is as far as it went. So that scares me, you know? O: Yeah, and that is very understandable.
Negative response *n* = 29 (19%)	P: This is a little more complicated than I thought. O: Oh, this is very complicated. This is a complicated – P: I thought that since it is such an early stage, that basically I would have a lumpectomy, and I would not need radiation. O: Right, so whatever your source of information was, it is not correct.

Abbreviations: O, Oncologist; P, Patient.

#### 2.2.4 Provider interruptions

Provider interruptions (RQ4 and RQ5) were considered when the oncologist's speaking turn cut patients off mid‐sentence or spoke over to the extent that patients could not be understood. This could occur at any moment in the conversation. Most consultations (76.5%) had ≥3 instances of the provider interruptions. Two appointments had no instances of provider interruptions; one appointment had 15. To account for varying appointment lengths, the number of interruptions was divided by the consultation length for a rate of provider interruptions. To account for varying patient participation, patient word count was divided by interruption rate to create a rate of provider interruptions as a function of participation.

### 2.3 Analyses

Initial analyses included bivariate correlations with Bonferroni‐adjusted significance levels across model variables. Hypothesized relationships among study variables were tested using two‐tailed *t*‐tests. Differences between patients with high versus low anxiety were compared on all study variables using *χ* ^2^ and two‐tailed *t*‐tests. Path analysis assessed overall fit of the proposed model. Model fit was assessed using a combination of fit statistics including *χ* ^2^, root mean square error of approximation (RMSEA), confirmatory fit index (CFI), and standardized root mean square residual (SRMR). Good fit was considered at RMSEA <0.06, CFI >0.95, and SRMR <0.08.^34^ Linear regression modeling was conducted using response types (missed, positive, or negative) as predictors and post‐appointment uncertainty as the dependent variable. The three potential response types (missed, positive, or negative) were treated as separate variables in the regression model, with separate dummy variables for each. A dummy variable of 1 was treated as the presence of that response type (missed, positive, or negative), and 0 was treated as the absence of that response type. Data were analyzed using STATA MP (version 17.0).

## 3 RESULTS

### 3.1 Participants, demographics, and descriptive statistics

Basic demographic information (age, education, race, marital status, and income) was collected (see Table 2). Consultation lengths ranged from 9.3 to 60.7 min (*M* = 27.4 min). Patients with higher levels of anxiety did not have more pre‐appointment uncertainty (*p* = 0.94) but were interrupted at a higher rate (*p* = 0.04) than those without anxiety. Pairwise correlation of model variables with Bonferroni corrections revealed only one statistically significant correlation; pre‐appointment uncertainty was correlated with post‐session uncertainty (*p* <0.01). Although moderate correlations were present (i.e., >0.25), no other relationships were significant (see Table 3).

**TABLE 2:** Demographic information and descriptive statistics.

	Total *N* = 47	Low anxiety score *n* = 35	High anxiety score *n* = 12	*p‐*value
Age—Mean (SE)	53.89 (1.58)	54.37 (1.73)	52.81 (3.40)	0.42^a^
Education—frequency				
High school or less	12	9	3	0.10^b^
2 years of college	8	7	1	
4 years of college	19	11	8	
Advanced degree	8	8	0	
Race/ethnicity—frequency				
White/Caucasian	39	31	8	0.16^b^
Black/African American	2	1	1	
Hispanic	2	0	2	
Asian	4	3	1	
Income—frequency				
$0–75,000	20	15	5	0.93^b^
$76,000–200,000+	23	17	6	
Marital status				
Single and never married	4	2	2	0.32^b^
Single and divorced/widowed	14	12	2	
Legally married or domestic partnership	29	21	8	

Abbreviation: SE, standard error.

^a^ One‐way analysis of variance with Bonferroni‐adjusted significance levels.

^b^ Chi‐squared test.

**TABLE 3:** Descriptive statistics and correlations between model variables.

Variable	M	SD	***α***	Correlations				
				(1)	(2)	(3)	(4)	(5)
Pre‐appointment anxiety	8.06	4.17	0.89	–				
2 Pre‐appointment uncertainty	15.25	5.57	0.84	0.30	–			
3 Post‐appointment uncertainty	11.82	3.82	0.80	0.28	0.59*	–		
4 Rate of empathic openings	0.11	0.08	–	0.12	0.25	−0.09	–	
5 Rate of interruptions	4.81	3.36	–	−0.20	0.06	−0.02	0.19	–

Abbreviations: *⍺*, Cronbach's alpha; M, mean; SD, standard deviation.

* *p* < 0.01.

### 3.2 Path modeling (H1, H2, RQ1, RQ2, RQ4, and RQ5)

Path modeling was performed to test whether the theoretical model in Figure 1A was supported. The proposed model achieved excellent data fit (*χ* ^2^ (4) = 4.29, *p* = 0.37; RMSEA = 0.04; CFI = 0.99; SRMR = 0.07) (see Figure 1B). The path from anxiety was positively associated with pre‐appointment uncertainty (*p* = 0.03); H1 supported. Pre‐appointment uncertainty was positively associated with post‐appointment uncertainty (*p* <0.01) such that more uncertainty pre‐appointment was associated with more uncertainty post‐appointment (H2 supported). The path from pre‐appointment uncertainty was positively associated with the rate of empathic openings (*p* = 0.02) such that more uncertainty was related to more empathic opportunities (RQ1). The path from empathic openings was negatively associated with post‐appointment uncertainty such that a higher rate of empathic openings was related to less post‐appointment uncertainty (RQ2), though this was not significant (*p* = 0.06). The paths from pre‐appointment uncertainty to provider interruptions (RQ4) and from provider interruptions to post‐appointment uncertainty (RQ5) were not statistically significant (*p* = 0.58 and 0.83, respectively).

### 3.3 Regression modeling (RQ3)

Linear regression modeling of post‐appointment uncertainty explored the possible influence of provider responses to empathic openings on post‐appointment uncertainty (RQ3). Results revealed no significant predictors, though positive responses were trending significant (*β* = −32.21, *p* = 0.06) such that a higher rate of positive responses to empathic openings was associated with lower levels of post‐session uncertainty. Missed opportunities (*β* = 4.21, *p* = 0.70) and negative responses (*β* = −19.96, *p* = 0.19) were not significant predictors. Overall, the regression model provided minimal explanatory power (*F* (3, 46) = 1.85, *p* = 0.15), predicting 10.76% of the variance in the response variable (*R* ^2^ = 0.11).

## 4 DISCUSSION

This study combined patient survey data and videorecorded medical interactions to consider patient perceptions and patient and physician behaviors when evaluating how pre‐appointment anxiety and pre‐appointment uncertainty affect patient and provider communication in breast cancer surgical consultations. The pre‐ and post‐appointment survey design supports the proposed directionality of patient and provider behaviors on patient outcomes. Findings highlight four noteworthy results. First, results supported the hypothesized relationships of UIT such that anxiety (*cognitive capacity*) predicted illness uncertainty. Specifically, pre‐appointment anxiety was associated with higher levels of pre‐appointment uncertainty. Second, increased uncertainty contributed to more patient empathic openings per minute. In other words, more anxious breast cancer patients arrived at their surgical consultation with more uncertainty about their illness and offered more statements about their emotions. This adds to extant literature that describes differences in patient participation as dependent on their psychological well‐being.^22^ Knowing that patients who arrived to their appointment with more anxiety had more uncertainty, these findings support emotion statements as a patient communication behavior that may have a positive effect on well‐being independent of provider communication. Third, although not statistically significant (*p* = 0.06), provider response to patient empathic openings is a fruitful avenue for additional investigation. Fourth, anxious patients experienced more provider interruptions, but this did not significantly impact patient anxiety.

### 4.1 Uncertainty in illness theory

This study applied pre‐appointment anxiety as an indicator of cognitive capacity, an antecedent of uncertainty appraisal. Unlike prior scholarship, results inform how cognitive capacity influences patient outcomes; specifically, pre‐appointment anxiety influenced pre‐appointment uncertainty, predicting increased patient empathic openings. Further, this study situates the logic of UIT as antecedent to actual patient–provider communication behaviors—a unique contribution to the body of work supporting UIT.

### 4.2 Patient empathic opportunities and provider response

Patient psychosocial states such as increased anxiety or uncertainty influence patient communication behavior.^22^, ^23^ This study documents how breast cancer patients with increased anxiety offer increased empathic opportunities. It is possible that empathic opportunities provide an opportunity for patients to indirectly request information or comfort that there are not comfortable asking outright. The empathic opening may allow for deniability of intent or may be more face‐saving^35^ than alternative forms of patient communication.

Providers' responses to patient talk can influence patient psychological outcomes. Although not significant, results suggest that positive, affirming provider response to patient empathic openings can promote reduced patient uncertainty. This supports prior work that identifies provider affective and supportive communication as predictive of beneficial patient outcomes such as satisfaction.^36^ Given the small sample size, future work should replicate these constructs to further examine the impact of varying provider response on patient uncertainty.

### 4.3 Provider interruptions

Patients with high anxiety experienced more provider interruptions per minute despite having no difference in the amount of patient participation. This may indicate a subtle difference in patient assertiveness when anxiety levels are high before medical appointments, aligning with the limited research available on this topic. For example, in family medicine contexts, higher levels of pre‐appointment patient anxiety predict higher levels of provider information‐giving^37^ and individual differences in breast cancer patient characteristics have been associated with different communication patterns during appointments.^38^ Future research should continue to investigate how patient psychological state is associated with varying provider communication patterns.

Contrary to expectation, there was not a significant association between provider interruption and patient outcomes. It is possible that the provider communication behavior of interruptions are associated with different patient outcome variables.^39^ An alternative explanation is that interruptions vary in form; some serve to overpower or disrupt the other's speech while others demonstrate a collaboration in speech.^39^, ^40^ Additional work may further investigate how provider communication behavior that may be deemed “problematic” may serve multiple communication purposes.

### 4.4 Study limitations

Along with the strengths above, there are important study limitations to consider. Although this study included recorded data from 51 patients, a limitation was the inclusion of one provider. We cannot conclude whether patterns seen in this study generalize to surgical oncology, early breast cancer appointments, oncology care more broadly, or if it is unique to this provider. Other oncologists may have different patterns of communication that could influence how these findings generalize across providers. Next, study participants were largely racially homogenous, limiting generalizability across demographic samples. As communication patterns can vary by patient demographic and concordance with clinicians,^41^ future research should recruit more diverse samples. Further, given the limited sample size of this study, detailed comparisons of model constructs across demographic variables were not sufficiently powered. Future studies should expand the current efforts by including multiple clinicians and replicating the methods within a population of patients of diverse sociodemographic identities. Despite these limitations, this study is expected to contribute to the broader literature on anxiety, illness uncertainty, and patient–provider communication in a breast cancer context.

### 4.5 Clinical implications

Results indicate breast cancer patients with anxiety pre‐appointment are at‐risk for more illness uncertainty and are more likely to explicitly provide empathic opportunities. This supports the need for added attention to empathic opportunities to not only address patients emotionally, but to also assess whether a patient may be at higher risk to have preexisting anxiety. Additionally, results provide evidence for the effects of positive provider responses to empathic openings. Although not significant in our data, positive responses to empathic openings trended towards significantly predicting a reduction of post‐appointment uncertainty. Because reduction of breast cancer patient uncertainty is associated with better psychological outcomes,^17^ this finding may have implications for patients beyond the initial interaction. Future research should clarify the effects of providers positively responding to empathic openings to address patient uncertainty versus the effects of providing an empathic opening on its own (i.e., indicating feeling comfortable with the provider).

## 5 CONCLUSIONS

The content of breast cancer surgical consultations (i.e., providing surgical treatment options and education) has been shown previously to help reduce patient anxiety.^42^ However, patient anxiety *pre*‐*appointment* has had limited investigation as an antecedent to patient–provider communication. Recognizing patients' psychological well‐being remains critical for all oncology care providers. Awareness of patient pre‐appointment anxiety can guide providers to invite more empathic opportunities and be cautious of their own communication behaviors. Results provide new understanding for how patients' pre‐appointment anxiety influences effective patient–provider communication and post‐appointment uncertainty. Assessing the psychological well‐being of breast cancer patients is crucial for identifying those at high risk of negative health outcomes and providing holistic care.

## AUTHOR CONTRIBUTIONS

**Elizabeth A. Broadbridge:** Conceptualization (lead); formal analysis (lead); investigation (equal); methodology (lead); visualization (lead); writing – original draft (lead). **Maria K. Venetis:** Conceptualization (supporting); investigation (equal); methodology (supporting); supervision (lead); writing – review and editing (lead).

## CONFLICT OF INTEREST STATEMENT

The authors declare no competing interests.

## Data Availability

Data sharing is not applicable to this article as no new data were created or analyzed in this study.
